# Behavioral Prevention Regarding Sexually Transmitted Infections and its Predictors in Women

**DOI:** 10.5812/ircmj.18346

**Published:** 2014-08-05

**Authors:** Azizeh Farshbaf-khalili, Mahnaz Shahnazi, Hanieh Salehi-pourmehr, Fatemeh Faridvand, Zoleikha Asgarloo

**Affiliations:** 1 Faculty of Nursing and Midwifery, Tabriz University of Medical Sciences, Tabriz, IR Iran; 2 Research Center of National Public Health Management (NPMC), Tabriz University of Medical Sciences, Tabriz, IR Iran

**Keywords:** Behavior, Prevention, Oral Sex, Infections, Women, Iran

## Abstract

**Background:**

Sexually transmitted infections (STIs) have a major negative impact on sexual and reproductive health globally. The most effective way to avoid STIs is to abstain from sexual contact or having sex only in a long-term, mutually monogamous relationship with an uninfected partner.

**Objectives:**

This study aimed to determine behavioral prevention regarding STIs.

**Patients and Methods:**

This analytic cross-sectional study was performed on 584 women aged 15-49 referring to health care centers of Tabriz-Iran in 2013 through multistage cluster sampling. Data collecting tool was a questionnaire which its validity and reliability were determined previously. Descriptive and inferential statistics (independent T-test, One-way ANOVA, and univariate and multivariate analyses) were used to analyze the data using SPSS 17. P < 0.05 was considered significant.

**Results:**

The mean ± SD score of behavioral prevention was 20.57 ± 2.8 ranging from 0 to 24. The weakest behavioral prevention was lack of consistent use of condom by husband during sex. The mean ± SD score of individuals’ awareness about STIs was as low as 17.08 ± 7.42. Multivariate analysis adjusting potential confounders showed a significant association between behavioral prevention and awareness, education, sex only with husband, anal, oral, and anal-oral sex.

**Conclusions:**

Prevention and care of sexually transmitted diseases are interventions able to promote public health. It is necessary to solve different factors affecting rapid spread of STDs and their transmission through an effective behavioral preventive plan.

## 1. Background

Sexually transmitted infections (STIs) have a major negative impact on sexual and reproductive health globally (1). In developing countries, STIs and their complications are classified as first five diseases that adults are seeking health care for them (1, 2). In total, 499 million new cases of curable STIs occur in adults aged 15-49 years around the world annually (1). STI is a public health problem (2) in most countries, especially developing countries and often affects young people (3) and is defined as a type of infection transmitted from person to person during sexual contact (4). Infection with special types of Human Papilloma Virus (HPV) can predispose genital cancer especially cervical cancer in women (1, 5). STIs are mainly transmitted by sexual contact with an infected person or from mother to child, but they can be transmitted by shared needles in injecting drug users (IDUs) (3). Early sexual activity leads to the increase of STDs (3, 6). In general, the probability of initial spread of any STI within a population depends on the rate of sexual exposure, probability of transmission per exposure and the duration that an infected person can transmit the infection (7, 8). Complications of STDs include chronic pain in abdomen, infertility in women, infertility and urinary stricture disease in men, miscarriage, ectopic pregnancy, genital cancer especially cervical cancer, stillbirth, congenital anomalies and newborn death, woman’s death by infection, neonatal eye infections and increasing the risk of HIV transmission (9, 10). STDs may cause serious problems in men (11). The risk of infection with STDs can be decreased by knowing sexual partners and restricting the number (1, 12), using latex condom in every vaginal, oral, and anal sex, avoiding risky sexual practices such as sexual acts that tear or break the skin leading to higher risk of STDs and finally immunization (13). Avoiding sexual intercourse is the only definite way to prevent pregnancy and STIs (12, 14). According to the statistics, up to 2012, 24290 individuals with HIV/AIDS were registered in Iran, of whom 90.8% were male and 9.2% were female. Totally, 46.4% of patients with HIV aged 25 to 34 at the time of getting HIV. Causes of HIV infection in 2011 were injecting 62.5%, sexual contact 21.2%, and transmitting from mother to child 3.9%. The route of transmission was not clear in 12.4% of people, that transmission via sexual contact is increasing (14). Although the number of people with AIDS was low in Iran, WHO reported that AIDS is seriously increasing in this country. The number of women with AIDS is increasing. More than 90% of women with HIV are infected through their husbands (14, 15). There are some problems in controlling STDs including difficulty in changing sexual behaviors formed according to the culture of each region and affected by religion, being personal and very deep subject, and also difficulty in discussing about sexual relations (12). It is necessary to solve various factors affecting rapid spread of STDs and their transmission through an effective behavioral preventive plan. The only vaccine for HIV is “behavioral prevention” (14). Prevention and care of STD promote public health. UNAIDS and WHO recommend to employ and develop high priority plans to achieve this goal. They also believe that every country should have a program for the prevention of STDs that should be integrated and fully coordinated with national AIDS programs (16). Preventive interventions have three components as follows:

- primary prevention, mainly directed towards uninfected people;
- secondary prevention, which includes early detection of infection to offer early prevention and therapeutic services to both acutely and chronically infected patients, to reduce their risky behaviors to decrease STI rates and HIV transmission to others and treat them as indicated; and
- tertiary prevention, which involves targeting patients with chronic infection to reduce their death and disability levels by using antiretroviral therapy and enabling partial immune reconstitution (17).

Behavioral prevention is effective with different age groups and at different levels of intervention when prevention program has a theoretical basis. Behavioral preventive interventions can be implemented in a community to address all of the factors associated with rapid development of an epidemy. Behavioral prevention is cost-effective and can be performed in communities with limited resources (18).

## 2. Objectives

This study was performed to determine behavioral prevention regarding STIs and its predictors in women in Iran.

## 3. Patients and Methods

This was an analytic cross-sectional study. The study population consisted of all women referring to health centers of Tabriz in 2013. Inclusion criteria were women referring to health centers for health care with records, having literacy of more than elementary school, willingness to participate in the research, not being single, and age range of 15 to 49. Exclusion criteria were individuals’ refusal from participation. The sample size was estimated 384 individuals using the formula for calculating proportion P = 50%, z = 1.96, and d = 0.05 and considering Design Effect = 1.5, finally it was calculated 584. Sampling was performed in two stages as multistage cluster sampling method. Randomly, 22 centers and stations (9 centers and 13 stations) were selected among 27 centers and 38 health stations. In the next stage, regarding the sample size, 25-30 health records were randomly selected among all the records of women. Patients were explained about the study by phone call and invited for an appointment. Sampling in both stages was performed randomly. We selected 621 eligible women at first, of whom 37 declined to participate and 584 consented. Data collecting tool was a researcher-regulated questionnaire designed according to information in available guidelines (1, 10, 12, 16). This questionnaire consisted of two parts: the first part included 12 questions about behavioral prevention regarding STIs and the second part was about factors affecting behavioral prevention containing 11 about sociodemographic characteristics and nine about history of midwifery and reproduction, the 37 questions about awareness, and 19 risk factors questions. Content and face validity were evaluated to determine the validity of the questionnaire. Ten faculty members evaluated the questionnaire and some modifications were made according to their feedbacks. CVI (Content Validity Index) and CVR (Content Validity Reliability) indices were 0.81 and 0.72, respectively. Test and re-test method was implemented on 30 subjects to determine the reliability (intraclass correlation coefficient, ICC) and internal consistency (Cronbach’s α coefficient). ICC (CI: 95%) and Cronbach’s α were 0.81 (0.73-0.88) and 0.8, respectively. Required permission was taken from the ethical committee of Tabriz University of Medical Sciences. Participants completed the questionnaire after the researcher explained the study objectives and differences between natural secretions of genital system and infections. Before data collection, a written consent was obtained from all participants. Voluntariness and confidentiality of the information were emphasized. In behavioral prevention, score 2 was assigned to “always”, score 1 to “sometimes”, and score 0 to “never”, then the total score was calculated. The minimum possible score of behavioral prevention was zero and the maximum was 24. To identify the level of awareness, score 1 was given to “right” answers and zero was given to “wrong” or “I do not know” answers, then the total score of each individual was calculated. Knowledge score below 12 was considered as weak, 13-24 as average and 25-37 as good. Data was analyzed using SPSS software (ver.17). Descriptive statistics were used to examine the frequency distribution, mean and CI 95% of the mean. To answer the questions of the research, Independent T-test, Mann-Whitney, Kruskal-Wallis and One-way ANOVA were used and to control confounding factors and identify their effects univariate and multivariate statistics were used. Before conducting multivariate analysis, the assumptions of the regression were studied, including the normality of the residuals, the homogeneity of the residual variance, the multicollinearity of independent variables, and the independence of the residuals. Variables with P < 0.1 entered the model. P < 0.05 was considered significant. This study was approved by the Research Deputy of Tabriz University of Medical Sciences, Tabriz, Iran (code: 91101).

## 4. Results

Most subjects aged 20 to 29 years. Most participants and their husbands had diploma. Most of them were housewives. Most of them had sufficient income. There was a significant association between the score of behavioral prevention and age, education, occupation and income. Individuals younger than 20 years had the least score of behavioral prevention. The score of prevention was higher in participants with high education (both herself and her husband), with occupation outside the home and sufficient income (Table 1).

**Table 1.** Association of Sociodemographic Characteristics With Behavioral Prevention^a^

Socio-Demographic Characteristics	Frequency	Behavioral Prevention^b^	P Value^c^
**Age group**			0.04
< 20	19 (3.3)	19.62 ± 2.83	
20-29	327 (56.1)	20.44 ± 2.99	
30-39	198 (34)	20.96 ± 2.45	
40-49	39 (6.6)	20.04 ± 2.73	
**Education level**			< 0.001
Primary school	63 (10.8)	18.81 ± 2.85	
Secondary school	85 (14.6)	20.33 ± 2.65	
High school	63 (10.8)	19.95 ± 3.38	
Diploma	282 (48.3)	20.81 ± 2.69	
Academic	91 (15.6)	21.69 ± 2.15	
**Employment status**			0.025
Housewife	525 (90.2)	20.54 ± 2.82	
Working at home	21 (3.6)	19.59 ± 2.98	
Working out home	36 (6.2)	21.59 ± 2.39	
**Income**			0.005
Sufficient	330 (59.4)	20.83 ± 2.76	
Insufficient	226 (40.6)	20.16 ± 2.74	
**Husband’s education**			< 0.001
illiterate	8 (1.4)	17.87 ± 2.29	
Primary school	70 (12)	20.09 ± 2.57	
Secondary school	138 (23.7)	20.24 ± 3.06	
High school	70 (12)	20.56 ± 3.21	
Diploma	192 (33)	20.59 ± 2.63	
Academic	104 (17.9)	21.57 ± 2.19	

^a^ Data are presented as No. (%) or Mean ± SD.

^b^ The minimum score of behavioral prevention was zero and the maximum was 24.

^c^ P < 0.05.

Regarding the risk factors, almost a half of the participants had a history of genital system infection. There was a statistically significant association between the score of behavioral prevention and sex only with husband, oral, anal, and vaginal-oral sex, and husband’s trip to abroad alone (Table 2).

**Table 2.** Frequency of Risk Factors and Their Association With Behavioral Prevention^a^

Risk Factors	Yes	No	Don’t Know	P Value
**Have you ever had a genital tract infection (vagina, uterus and pelvis)?**	263 ± 45.7	313 ± 54.3	-	0.2
**Do you have a history of going to prison?**	1 ± 0.2	580 ± 99.8	-	0.26
**Does your partner have a history of going to prison?**	9 ± 1.6	553 ± 95.5	17 ± 2.9	0.32
**Does your husband have traveled abroad alone?**	67 ± 11.7	503 ± 87.5	5 ± 0.9	0.005^b^
**Are you a drug addict?**	1 ± 0.2	578 ± 99.8	-	0.53
**Does your husband have a drug addiction?**	5 ± 0.5	57 ± 98.6	3 ± 0.9	0.14
**Do you have a history of blood transfusion?**	4 ± 0.7	572 ± 99.1	1 ± 0.2	0.31
**Does your husband have a history of blood transfusion?**	12 ± 2.1	551 ± 95.2	16 ± 2.8	0.28
**Do you have warts, blisters or sores on the genitalia, anus, or surrounding areas?**	11 ± 1.9	569 ± 98.1	-	0.48
**Does your husband have warts, blisters or sores on the genitalia, anus, or surrounding areas?**	8 ± 1.4	571 ± 98.6	-	0.73
**Do you have oral, anal, and vaginal-oral sex?**	40 ± 6.9	515 ± 88.9	24 ± 4.1	< 0.001^b^
**Do you have sex only with husband?**	556 ± 96.2	22 ± 3.8	-	< 0.001^b^

^a^ Independent T-test, One-way ANOVA, Mann-Whitney and Kruskal-Wallis were used. Data are presented as mean ± SD.

^b^ P < 0.05.

The mean ± SD score of behavioral prevention was 20.57 ± 2.8 ranging from 0 to 24. The weakest behavioral prevention items were not continuous use of condom by husband during sexual relationship (83.2%) and not seeking rapid diagnosis and treatment by man (36.7%) and woman (28.7%) in case of STD (such as genital wart or herpes, hepatitis B, AIDS, etc.). The strongest behavioral preventions were respectively; not having sex with someone with symptoms of STDs (99%), not having sexual relationship with injected drug users (98.6%), not having sex with someone who has recently been in prison (98.3%), and having sex with only one person (98.3%) (Table 3).

**Table 3.** Behavioral Prevention Regarding Sexually Transmitted Infections^a,b^

Preventive Behavior	Always	Sometimes	Never
**Having sex with one person**	574 (98.3)	3 (0.5)	1 (0.2)
**Using condom by husband or partner during sex**	97 (16.8)	204 (35.3)	277 (47.9)
**Not having sex with someone with symptoms of STDs**	573 (99)	2 (0.3)	4 (0.7)
**Not having sex with someone who has sex with others **	558 (98.1)	3 (0.5)	8 (1.4)
**Having sex only with husband**	556 (96.2)	3 (0.5)	19 (3.3)
**Not having sex with injected drug users**	569 (98.6)	4 (0.7)	4 (0.7)
**Not having sex with someone who has recently been in prison**	567 (98.3)	9 (1.6)	1 (0.2)
**Not having oral, anal, and vaginal-oral sex**	468 (81.1)	101 (17.5)	8 (1.4)
**Seeking rapid diagnosis and treatment in case of STD **	408 (71.3)	55 (9.6)	109 (19.1)
**Seeking rapid diagnosis and treatment by husband in case of STD**	353 (62.1)	96 (16.9)	119 (21)
**Regular intake of necessary drugs in the case of STD**	441 (77.4)	79 (13.9)	50 (8.8)
**Doing necessary follow-up to ensure complete cure of infection**	406 (71.1)	111 (19.4)	54 (9.5)

^a^ Data are presented as No. (%).

^b^ Range of scores was 0 to 24.

The mean ± SD score of individuals’ awareness about STIs was as low as 17.08 ± 7.42 ranging 0-37. There was a significant statistical association between individuals’ awareness level and the score of behavioral prevention (P < 0.001). By increasing the awareness level, the score of behavioral prevention promoted significantly. Fourteen of 37 questions about awareness were related to knowledge about HPV; the median (min, max) of the individuals’ score was very low as 3 (0, 13). Multivariate analysis adjusting for potential confounders showed a significant statistical association between behavioral prevention and awareness score (β = 0.48, 95% CI = 0.02-0.08). Therefore, for every unit increase in awareness score, behavioral prevention increased 0.48 units. In addition, there was a significant statistical association between behavioral prevention and educational level (β = -1.93, 95% CI = -2.98-0.87), "sex only with husband" (β = 3.65, 95% CI = 2.51-4.79), and "anal, oral and anal-oral sex" (β = 1.776, 95% CI = 0.948-2.604) (Table 4).

**Table 4.** Predictive Value of Potential Confounding Variables for Behavioral Prevention Using General Linear Model^a^

Variables	Adjusted		Unadjusted		R^2^
	β (CI 95%)	P Value	β (CI 95%)	P Value	
**Family income**					0.014
Inadequate	Ref		Ref		
Adequate	0.410 (-0.035-0.855)	0.07	0.672 (0.204-1.139)	0.005	
Education					0.078
Academic	Ref		Ref		
Primary school	-1.926 (-2.979-0.872)	< 0.001	-2.888 (-3.764 to -2.012)	< 0.001	
Secondary school	-0.769 (-1.714-0.176)	0.11	-1.365 (-2.167 to -0.562)	0.001	
High school	-0.988 (-1.966 to -0.015)	0.04	-1.741 (-2.613 to -0.869)	< 0.001	
Diploma	-0.325 (-1.055-0.405)	0.38	-0.884 (-1.526 to -0.243)	0.007	
**Age**					0.014
< 20	Ref		Ref		
20-29	-0.105 (-1.57-1.36)	0.89	0.42 (-1.113-1.962)	0.58	
30-39	-0.183 (-1.381-1.015)	0.76	0.83 (-0.471-2.123)	0.21	
40-49	0.013 (-1.219-1.245)	0.98	1.35 (0.027-2.667)	0.046	
**Occupation**					0.013
Occupied out home	Ref		Ref		
Housewife	0.097 (-0.847-1.041)	0.94	-1.054 (-2.00 to -0.106)	0.03	
Occupied at home	-0.506 (-1.980 to- 0.969)	0.50	-1.998 (-3.509 to -0.488)	0.01	
**Wife education**					0.043
Academic	Ref		Ref		
Illiterate	-0.99 (-3.69-1.088)	0.35	-3.692 (-5.664 to -1.721)	< 0.001	
Primary school	-0.50 (-0.093-0.893)	0.92	-1.481 (-2.316 to -0.647)	0.001	
Secondary school	-0.319 (-1.118-0.480)	0.43	-1.322 (-2.020 to -0.624)	< 0.001	
High school	-0.318 (-1.200-0.564)	0.48	-1.007 (-1.838 to -0.176)	0.018	
Diploma	-0.477 (-1.159-0.205)	0.17	-0.977 (-1.631 to -0.323)	0.003	
**Husband trip to abroad alone**					0.018
Yes	Ref		Ref		
No	-1.13 (-3.68-1.43)	0.39	-3.677 (-6.201 to -1.152)	0.004	
Doesn’t know	-0.05 (-0.73-0.64)	0.89	-0.755 (-1.463 to -0.047)	0.037	
**Anal, oral, anal-oral sex**					0.038
Yes	Ref		Ref		
No	1.776 (0.948-2.604)	< 0.001	1.857 (0.967-2.746)	< 0.001	
Doesn’t know	0.912 (-0.378-2.203)	0.16	0.343 (-1.056-1.743)	0.63	
**Sex only with husband**					0.088
Yes	Ref		Ref		
No	3.650 (2.513-4.786)	< 0.001	4.321 (3.183-5.459)	< 0.001	
**knowledge**	0.48 (0.016-0.080)	0.003	0.097 (0.068-0.127)	< 0.001	0.06

^a^ R^2^ was 0.238 for all the adjusted variables.

## 5. Discussion

In this study, the weakest behavioral prevention items were; not continuous use of condom by husband (83.2%) and not looking for rapid diagnosis and treatment by man (36.7%) and woman (28.7%) in case of sexual disease. Hickey et al. studied 458 sexually active female students and found that despite low levels of condom use, most subjects did not consider themselves at risk of STIs (19). Kolahi et al. in their study on a female at-risk population showed that most subjects knew that proper condom use (78.1%) and having a single sexual partner (68.8%) can reduce the risk of infection. Of participants, 43.4% knew that an HIV-positive person can seem perfectly healthy (20). There are some data about sex workers in Iran within the WHO reports and according to mentioned reports, only 55% of Iranian sex workers reported the use of condom with their most recent clients (21). In the study of Ahmadnezhad et al. girls believed that AIDS is a preventable disease. Some believed that there is a vaccine available for prevention. Condom was not mentioned in any of the interviews and some of them did not know about it (22). In the interventional study of Sakha, there were significant improvements in attitude and the number of participants who reported preventive behaviors such as consistently use of condom (P < 0.001). The results showed that educational program was successful in increasing the participants' HIV- and AIDS-related knowledge and attitudes and decreasing their risky behaviors (23). In a cross-sectional study conducted in 2004 on 100 prisoner men, HIV high-risk behaviors decreased when subjects believed in the effectiveness of strategies designed to reduce the risk or seriousness of impact of the health condition (24). All these studies are in concordance with this study and showed that behavioral prevention programs should be performed to improve the awareness, attitude and behavior of people particularly those with low levels of risk perception for STIs. Shepherd et al. in their systematic review concluded that school-based behavioral interventions for prevention of STIs in young people can promote knowledge and increase efficacy, but interventions have no significantly effect on risky sexual behaviors or the rate of infection. Future researches should include long-term follow-up for employing healthier sexual behaviors and keeping it in adulthood (25). According to the guideline of CDC STD (The Centers for Disease Control and Prevention (CDC) Sexually Transmitted Disease (STD) Treatment Guidelines), controlling and preventing STIs are based on some mechanisms that contain education and consultation to affect preventive behavior, identification of new infections, treating present infections, evaluating, consulting and treating partners infected with STIs and providing vaccinations before sexual relationship (11). Regarding the increase of STDs in Iran, in addition to the mentioned issues, to pay attention to family units and values, provide housing to decrease the disruption to family life, occupation, education, religion, culture, age, gender, and etc. should be considered by statesmen and politicians. In this study, there was a significant statistical association between behavioral prevention and awareness score. As per a unit increase in the score of awareness, there was 0.48 unit increase in behavioral prevention. Besides, there was a significant statistical association between behavioral prevention and the level of education, sex only with husband and oral, anal and anal-oral sex. Behavioral prevention in people with university degrees was significantly better than those with elementary education; however, in people who had limited sexual relationship only with husband was better than non-limited relationships; also in individuals without anal, oral and anal-oral sex was better compared to individuals with such experiences. Here, the effect of sex only with husband and then educational level were higher than other variables when investigating all of them using univariate analysis without adjusting. In the study by Johnson et al. there was a positive association between information about sexual relationship and attitude toward using condom, and valuable health had a positive association with sexual knowledge and with attitude toward using condom and negative association with attitude toward sexual relationship (26). The result of Johnson's study indicated a positive association between health seeking behaviors and STIs prevention in concordance with our study. The American Academy of Family Physicians recommends counseling adolescents and adults about the risks of STDs and how to avoid them (13). Screening, treatment and follow-up of STIs are important parts of health-care providers’ duties for women (27). In this study, subjects were selected randomly from different regions and different social-economical levels. The predictors of STIs were determined in reproductive women. Low awareness of subjects about STIs especially HPV as a main cause of cervical cancer was found. It was performed according to active health-care records of married women aged 15-49 in Tabriz health centers and therefore, generalization of the results to other age groups should be undertaken with caution. It is recommended to conduct further studies on menopause and single people. Furthermore, a similar study can be performed in schools and universities. Studies on men can also be implemented. Investigating high-risk groups can also present useful information in this field. Other recommendation is presenting necessary educational interventions on STIs and investigating the effect of continuous and long-term education on behavioral prevention. Education and consultation about STIs should be a priority of our government. Establishing STIs and behavioral prevention clinics for education, screening, diagnosis and treatment with covering chastity homes and sex workers are important issues. The role of the mass media should not be ignored. Besides, easy access to condom, and screening, diagnostic and treatment facilities is helpful. Besides, cultural values preserving family respect and loyalty to spouse are of great importance, also government attention to issues such as providing easy marriage conditions for young people, and providing employment and eradicating poverty in the country are some essential parts of the plan.
